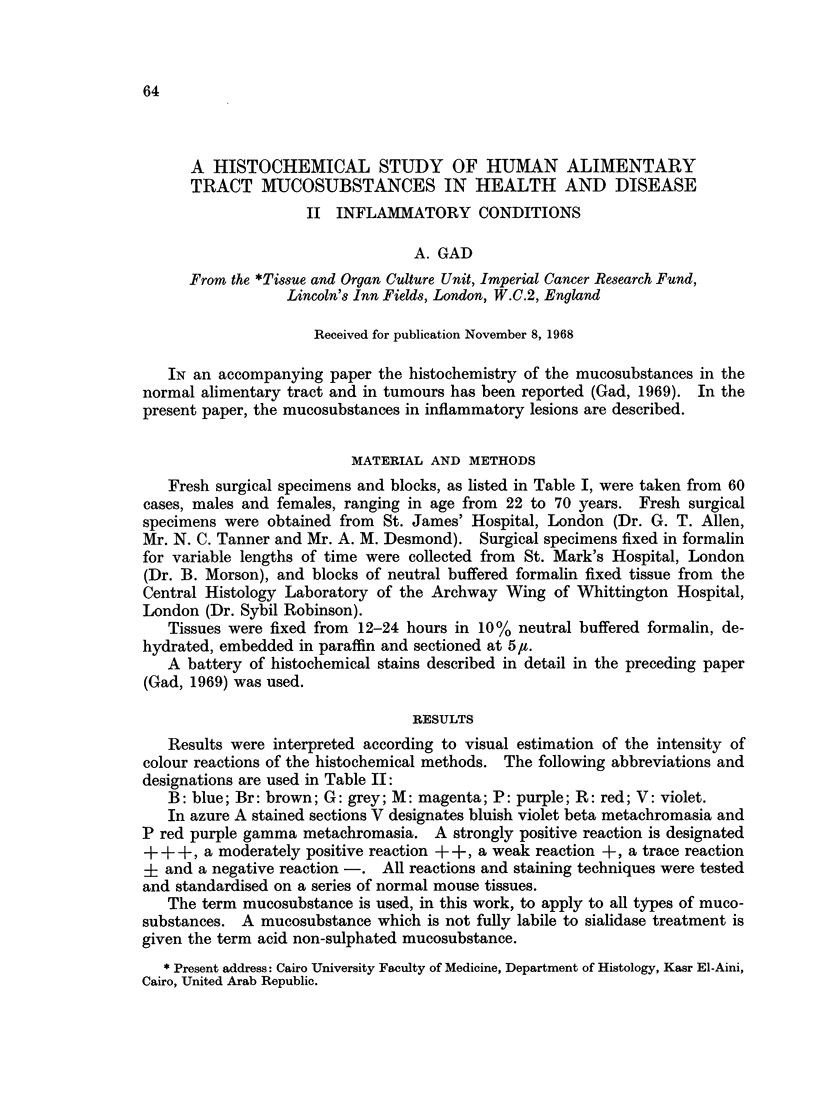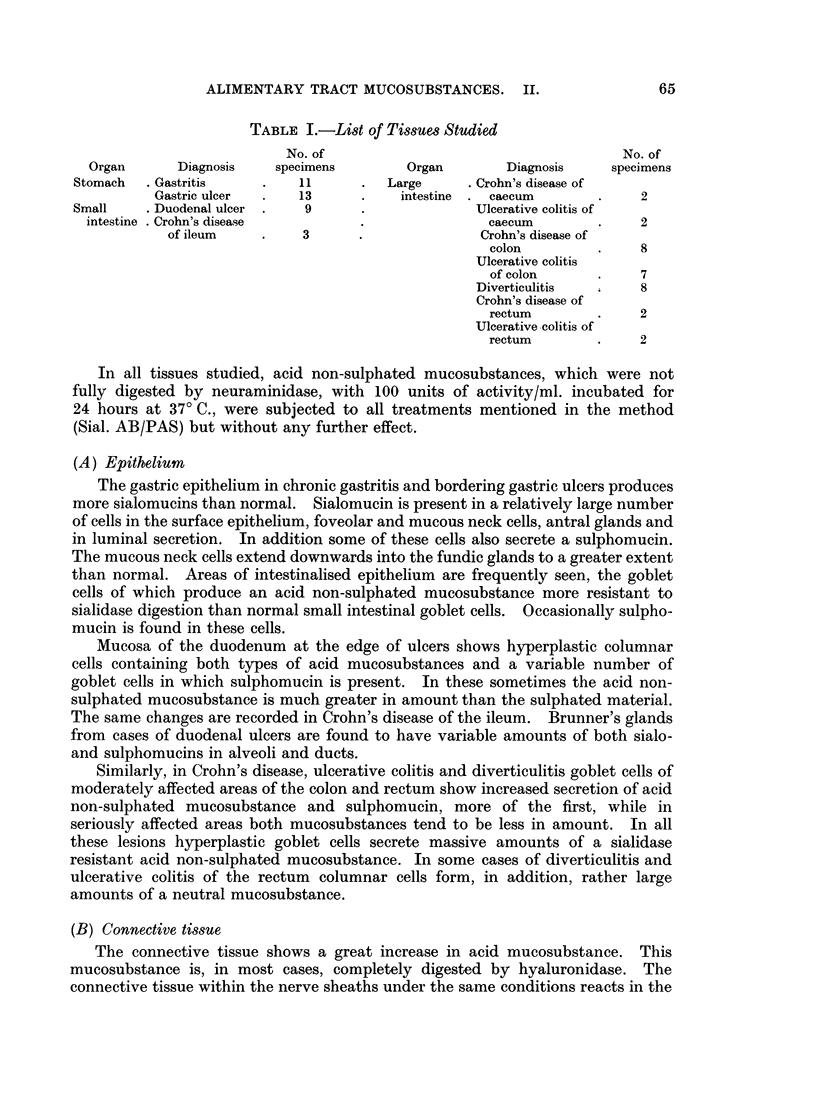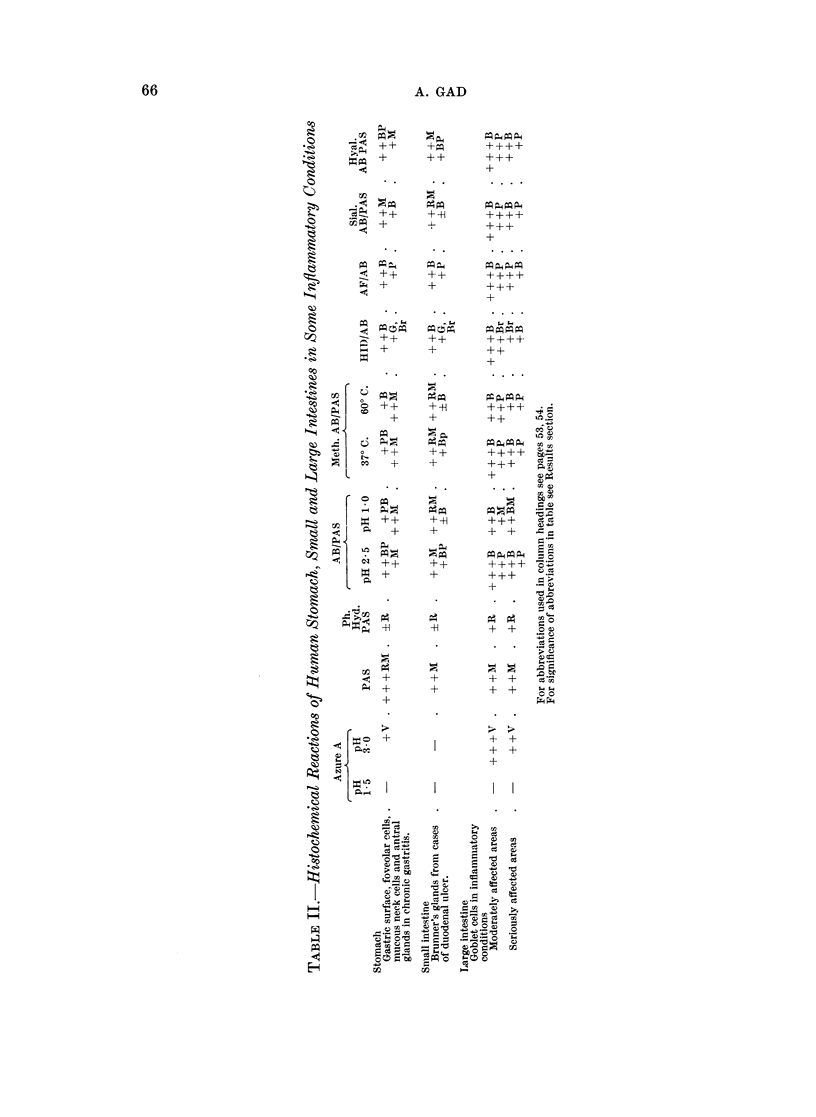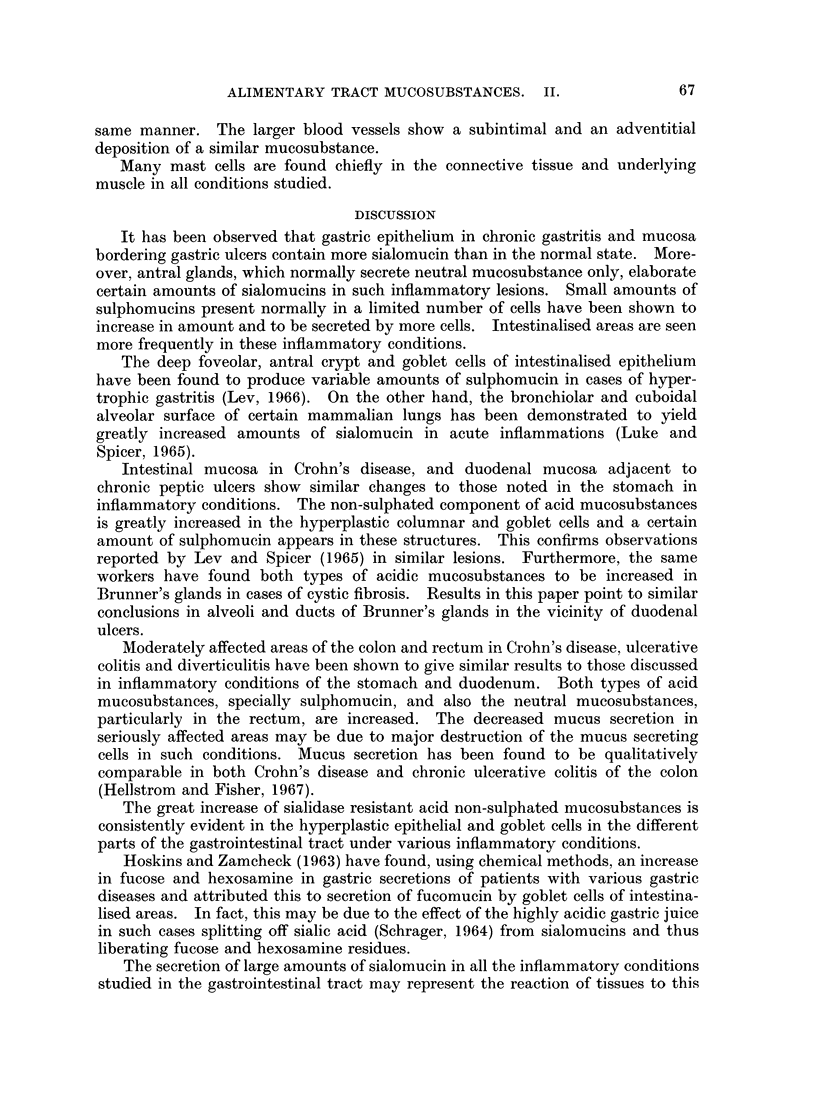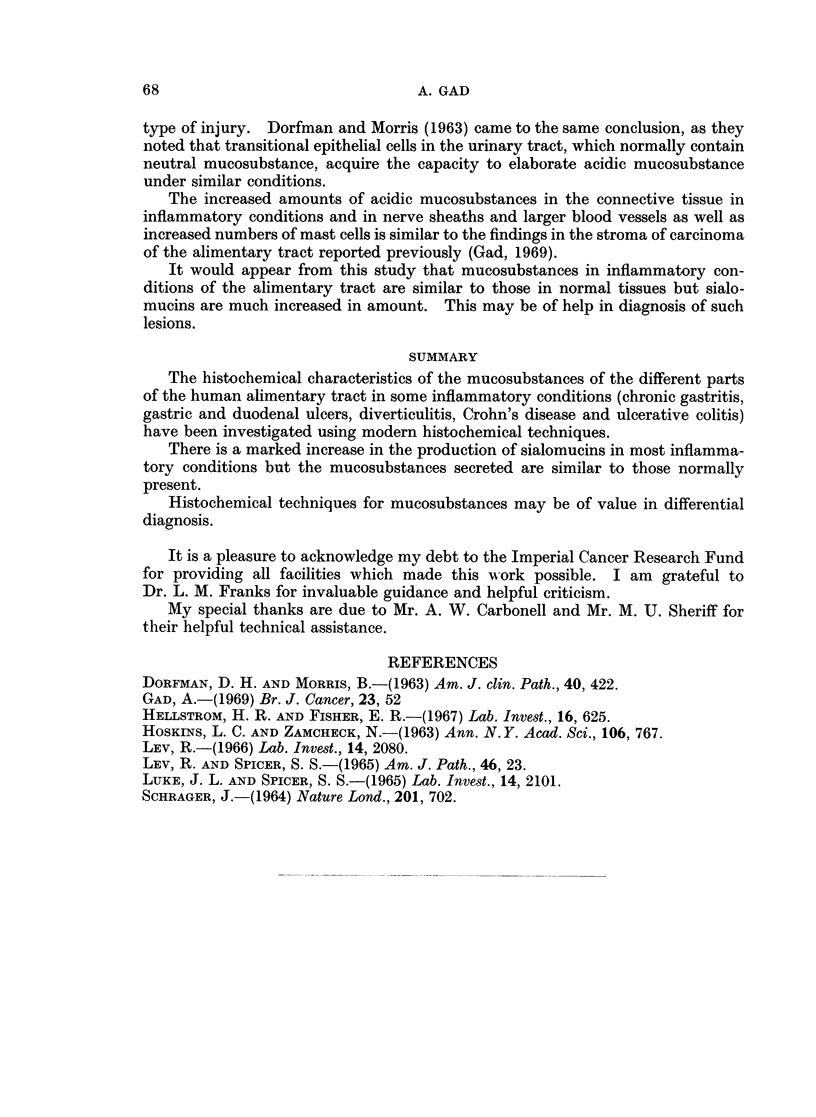# A histochemical study of human alimentary tract mucosubstances in health and disease. II. Inflammatory conditions.

**DOI:** 10.1038/bjc.1969.10

**Published:** 1969-03

**Authors:** A. Gad


### A HISTOCHEMICAL STUDY OF HUMAN ALIMENTARY TRACT MUCOSUBSTANCES IN HEALTH AND DISEASE

### II INFLAMMATORY CONDITIONS

A. GAD

*From the *Tissue and Organ Culture Unit, Imperial Cancer Research Fund, Lincoln's Inn Fields, London, W.C.2, England*

Received for publication November 8, 1968

In an accompanying paper the histochemistry of the mucosubstances in the normal alimentary tract and in tumours has been reported (Gad, 1969). In the present paper, the mucosubstances in inflammatory lesions are described.

### MATERIAL AND METHODS

Fresh surgical specimens and blocks, as listed in Table I, were taken from 60 cases, males and females, ranging in age from 22 to 70 years. Fresh surgical specimens were obtained from St. James' Hospital, London (Dr. G. T. Allen, Mr. N. C. Tanner and Mr. A. M. Desmond). Surgical specimens fixed in formalin for variable lengths of time were collected from St. Mark's Hospital, London (Dr. B. Morson), and blocks of neutral buffered formalin fixed tissue from the Central Histology Laboratory of the Archway Wing of Whittington Hospital, London (Dr. Sybil Robinson).

Tissues were fixed from 12–24 hours in 10% neutral buffered formalin, dehydrated, embedded in paraffin and sectioned at $5\mu$.

A battery of histochemical stains described in detail in the preceding paper (Gad, 1969) was used.

### RESULTS

Results were interpreted according to visual estimation of the intensity of colour reactions of the histochemical methods. The following abbreviations and designations are used in Table II:

B: blue; Br: brown; G: grey; M: magenta; P: purple; R: red; V: violet.

In azure A stained sections V designates bluish violet beta metachromasia and P red purple gamma metachromasia. A strongly positive reaction is designated $+++$, a moderately positive reaction $++$, a weak reaction $+$, a trace reaction $\pm$ and a negative reaction —. All reactions and staining techniques were tested and standardised on a series of normal mouse tissues.

The term mucosubstance is used, in this work, to apply to all types of mucosubstances. A mucosubstance which is not fully labile to sialidase treatment is given the term acid non-sulphated mucosubstance.

* Present address: Cairo University Faculty of Medicine, Department of Histology, Kasr El-Aini, Cairo, United Arab Republic.

TABLE I.—*List of Tissues Studied*

| Organ | Diagnosis | No. of specimens | Organ | Diagnosis | No. of specimens |
|---|---|---|---|---|---|
| Stomach | Gastritis | 11 | Large intestine | Crohn's disease of caecum | 2 |
| | Gastric ulcer | 13 | | Ulcerative colitis of caecum | 2 |
| Small intestine | Duodenal ulcer | 9 | | Crohn's disease of colon | 8 |
| | Crohn's disease of ileum | 3 | | Ulcerative colitis of colon | 7 |
| | | | | Diverticulitis | 8 |
| | | | | Crohn's disease of rectum | 2 |
| | | | | Ulcerative colitis of rectum | 2 |

In all tissues studied, acid non-sulphated mucosubstances, which were not fully digested by neuraminidase, with 100 units of activity/ml. incubated for 24 hours at 37° C., were subjected to all treatments mentioned in the method (Sial. AB/PAS) but without any further effect.

(*A*) *Epithelium*

The gastric epithelium in chronic gastritis and bordering gastric ulcers produces more sialomucins than normal. Sialomucin is present in a relatively large number of cells in the surface epithelium, foveolar and mucous neck cells, antral glands and in luminal secretion. In addition some of these cells also secrete a sulphomucin. The mucous neck cells extend downwards into the fundic glands to a greater extent than normal. Areas of intestinalised epithelium are frequently seen, the goblet cells of which produce an acid non-sulphated mucosubstance more resistant to sialidase digestion than normal small intestinal goblet cells. Occasionally sulphomucin is found in these cells.

Mucosa of the duodenum at the edge of ulcers shows hyperplastic columnar cells containing both types of acid mucosubstances and a variable number of goblet cells in which sulphomucin is present. In these sometimes the acid non-sulphated mucosubstance is much greater in amount than the sulphated material. The same changes are recorded in Crohn's disease of the ileum. Brunner's glands from cases of duodenal ulcers are found to have variable amounts of both sialo- and sulphomucins in alveoli and ducts.

Similarly, in Crohn's disease, ulcerative colitis and diverticulitis goblet cells of moderately affected areas of the colon and rectum show increased secretion of acid non-sulphated mucosubstance and sulphomucin, more of the first, while in seriously affected areas both mucosubstances tend to be less in amount. In all these lesions hyperplastic goblet cells secrete massive amounts of a sialidase resistant acid non-sulphated mucosubstance. In some cases of diverticulitis and ulcerative colitis of the rectum columnar cells form, in addition, rather large amounts of a neutral mucosubstance.

(*B*) *Connective tissue*

The connective tissue shows a great increase in acid mucosubstance. This mucosubstance is, in most cases, completely digested by hyaluronidase. The connective tissue within the nerve sheaths under the same conditions reacts in the

TABLE II.—*Histochemical Reactions of Human Stomach, Small and Large Intestines in Some Inflammatory Conditions*

| | Azure A | | | | AB/PAS | | Meth. AB/PAS | | | | | |
|---|---|---|---|---|---|---|---|---|---|---|---|---|
| | pH 1·5 | pH 3·0 | PAS | Ph. Hyd. PAS | pH 2·5 | pH 1·0 | 37° C. | 60° C. | HID/AB | AF/AB | Sial. AB/PAS | Hyal. AB PAS |
| Stomach<br>Gastric surface, foveolar cells, mucous neck cells and antral glands in chronic gastritis. | — | +V | +++RM | ±R | ++BP<br>+M | +PB<br>++M | +PB<br>++M | +B<br>++M | ++B<br>+G,<br>Br | ++B<br>+P | ++M<br>+B | ++BP<br>+M |
| Small intestine<br>Brunner's glands from cases of duodenal ulcer. | — | — | ++M | ±R | ++M<br>+BP | ++RM<br>±B | ++RM<br>+Bp | ++RM<br>±B | ++B<br>+G,<br>Br | ++B<br>+P | ++RM<br>±B | ++M<br>+BP |
| Large intestine<br>Goblet cells in inflammatory conditions<br>Moderately affected areas | — | +++V | ++M | +R | +++B<br>++P | ++B<br>+M | +++B<br>++P | ++B<br>++P | +++B<br>++Br | +++B<br>++P | +++B<br>++P | +++B<br>++P |
| Seriously affected areas | — | ++V | ++M | +R | ++B<br>+P | ++BM | ++B<br>+P | +B<br>+P | +Br<br>+B | ++P<br>+B | ++B<br>+P | ++B<br>+P |

For abbreviations used in column headings see pages 53, 54.
For significance of abbreviations in table see Results section.

same manner. The larger blood vessels show a subintimal and an adventitial deposition of a similar mucosubstance.

Many mast cells are found chiefly in the connective tissue and underlying muscle in all conditions studied.

### DISCUSSION

It has been observed that gastric epithelium in chronic gastritis and mucosa bordering gastric ulcers contain more sialomucin than in the normal state. Moreover, antral glands, which normally secrete neutral mucosubstance only, elaborate certain amounts of sialomucins in such inflammatory lesions. Small amounts of sulphomucins present normally in a limited number of cells have been shown to increase in amount and to be secreted by more cells. Intestinalised areas are seen more frequently in these inflammatory conditions.

The deep foveolar, antral crypt and goblet cells of intestinalised epithelium have been found to produce variable amounts of sulphomucin in cases of hypertrophic gastritis (Lev, 1966). On the other hand, the bronchiolar and cuboidal alveolar surface of certain mammalian lungs has been demonstrated to yield greatly increased amounts of sialomucin in acute inflammations (Luke and Spicer, 1965).

Intestinal mucosa in Crohn's disease, and duodenal mucosa adjacent to chronic peptic ulcers show similar changes to those noted in the stomach in inflammatory conditions. The non-sulphated component of acid mucosubstances is greatly increased in the hyperplastic columnar and goblet cells and a certain amount of sulphomucin appears in these structures. This confirms observations reported by Lev and Spicer (1965) in similar lesions. Furthermore, the same workers have found both types of acidic mucosubstances to be increased in Brunner's glands in cases of cystic fibrosis. Results in this paper point to similar conclusions in alveoli and ducts of Brunner's glands in the vicinity of duodenal ulcers.

Moderately affected areas of the colon and rectum in Crohn's disease, ulcerative colitis and diverticulitis have been shown to give similar results to those discussed in inflammatory conditions of the stomach and duodenum. Both types of acid mucosubstances, specially sulphomucin, and also the neutral mucosubstances, particularly in the rectum, are increased. The decreased mucus secretion in seriously affected areas may be due to major destruction of the mucus secreting cells in such conditions. Mucus secretion has been found to be qualitatively comparable in both Crohn's disease and chronic ulcerative colitis of the colon (Hellstrom and Fisher, 1967).

The great increase of sialidase resistant acid non-sulphated mucosubstances is consistently evident in the hyperplastic epithelial and goblet cells in the different parts of the gastrointestinal tract under various inflammatory conditions.

Hoskins and Zamcheck (1963) have found, using chemical methods, an increase in fucose and hexosamine in gastric secretions of patients with various gastric diseases and attributed this to secretion of fucomucin by goblet cells of intestinalised areas. In fact, this may be due to the effect of the highly acidic gastric juice in such cases splitting off sialic acid (Schrager, 1964) from sialomucins and thus liberating fucose and hexosamine residues.

The secretion of large amounts of sialomucin in all the inflammatory conditions studied in the gastrointestinal tract may represent the reaction of tissues to this type of injury. Dorfman and Morris (1963) came to the same conclusion, as they noted that transitional epithelial cells in the urinary tract, which normally contain neutral mucosubstance, acquire the capacity to elaborate acidic mucosubstance under similar conditions.

The increased amounts of acidic mucosubstances in the connective tissue in inflammatory conditions and in nerve sheaths and larger blood vessels as well as increased numbers of mast cells is similar to the findings in the stroma of carcinoma of the alimentary tract reported previously (Gad, 1969).

It would appear from this study that mucosubstances in inflammatory conditions of the alimentary tract are similar to those in normal tissues but sialomucins are much increased in amount. This may be of help in diagnosis of such lesions.

### SUMMARY

The histochemical characteristics of the mucosubstances of the different parts of the human alimentary tract in some inflammatory conditions (chronic gastritis, gastric and duodenal ulcers, diverticulitis, Crohn's disease and ulcerative colitis) have been investigated using modern histochemical techniques.

There is a marked increase in the production of sialomucins in most inflammatory conditions but the mucosubstances secreted are similar to those normally present.

Histochemical techniques for mucosubstances may be of value in differential diagnosis.

It is a pleasure to acknowledge my debt to the Imperial Cancer Research Fund for providing all facilities which made this work possible. I am grateful to Dr. L. M. Franks for invaluable guidance and helpful criticism.

My special thanks are due to Mr. A. W. Carbonell and Mr. M. U. Sheriff for their helpful technical assistance.

### REFERENCES

Dorfman, D. H. and Morris, B.—(1963) *Am. J. clin. Path.*, **40**, 422.
Gad, A.—(1969) *Br. J. Cancer*, **23**, 52
Hellstrom, H. R. and Fisher, E. R.—(1967) *Lab. Invest.*, **16**, 625.
Hoskins, L. C. and Zamcheck, N.—(1963) *Ann. N.Y. Acad. Sci.*, **106**, 767.
Lev, R.—(1966) *Lab. Invest.*, **14**, 2080.
Lev, R. and Spicer, S. S.—(1965) *Am. J. Path.*, **46**, 23.
Luke, J. L. and Spicer, S. S.—(1965) *Lab. Invest.*, **14**, 2101.
Schrager, J.—(1964) *Nature Lond.*, **201**, 702.